# Japan ECMOnet for COVID-19: telephone consultations for cases with severe respiratory failure caused by COVID-19

**DOI:** 10.1186/s40560-020-00440-9

**Published:** 2020-04-07

**Authors:** Japan ECMOnet for COVID-19

**Affiliations:** Department of Emergency and Critical Care Medicine, Shimane University, Ennya-cho 89-1, Izumo, Shimane Japan

**Keywords:** COVID-19, ECMO, Acute respiratory failure

## Abstract

Recently, the novel coronavirus disease 2019 (COVID-19) started spreading in Japan. Therefore, the number of patients with severe COVID-19 requiring extracorporeal membranous oxygenation (ECMO) is expected to increase. A working group has been formed to provide telephone consultation services for cases with severe respiratory failure caused by COVID-19 in Japan. During the first 13 days of the service, there were 12 consultations. For each consultation, we advised the patient on the initiation of ECMO and arranged transportation for patients with ECMO. Based on experience from the H1N1 influenza pandemic, transporting patients to experienced facilities is essential to obtain better outcomes.

**To the Editor**

The novel 2019 coronavirus-induced disease (COVID-19) has recently started spreading in Japan. According to the initial report from Wuhan in China, 4 of 138 hospitalized patients (2.8%) required extracorporeal membranous oxygenation (ECMO) [1]. As treatment for severe respiratory failure with ECMO requires specialized knowledge and training, the number of patients who present to a hospital for ECMO annually is positively associated with the survival rate of the patients [2]. It is also known that hospitalization at a center where health care professionals are well-trained on respiratory care results in improved outcomes [3].

Based on these findings, the Japanese Society of Intensive Care Medicine, the Japanese Association for Acute Medicine, the Japanese Society of Respiratory Care Medicine, and the PCPS/ECMO study group launched a COVID-19 ECMOnet working group in Japan. The group provides telephone consultations available 24 h a day for patients with severe respiratory failure, especially those who require ECMO. This group comprises 19 physicians who are well-trained ECMO specialists selected from all over Japan; initially, these specialists were mainly from the Kanto area. The consultations were initiated on February 15, 2020 with the Japanese Respiratory Society joining on February 20, and the Japanese Society for Infectious Disease joining on February 21. The telephone number was assigned to the members of this society. Furthermore, the Japanese Society of Intensive Care Medicine and the Japanese Association for Acute Medicine, registered in the Designated Medical Institutions for Infectious Diseases, compiled a registry system to review available beds for the ECMO run.

Between February 15 and 27, there were 12 consultations during the 12-day period. The mean age of the patients was 71.2, and the mean P/F ratio was 93.0. All patients were COVID-19 PCR positive. Nine of 12 patients consulted on the initiation of and general questions about ECMO. We recommended the initiation of ECMO for 3 cases: one was introduced to the ECMO at the local hospital, and second was introduced by ECMO specialists who came from an ECMO center and transported the patient to the ECMO center, and the last patient is currently arranging transportation to an ECMO center. Three of 12 patients had already had ECMO performed and consulted on further management. One patient consulted for secondary transport to an ECMO center; we discussed the indications and arranged transportation to the higher ECMO center.

The survival discharge rate for 2009 H1N1 influenza patients in Japan was only 35.7% [4], which, surprisingly, was much lower than that the 100% survival discharge rate at Karolinska University Hospital in Sweden [5]. There are three main reasons for this difference. First, the patients in Japan were not hospitalized at specialized hospitals with many facilities used V-V ECMO for the first time. Second, the diameter of the cannula used in Japan was small, resulting in insufficient blood flow. Third, the health care professionals who handled the patients were not well-informed about the general management during V-V ECMO; the main purpose of using ECMO is not oxygenation but lung rest. Mechanical ventilation which exceeds 1 week is not indicated before introducing ECMO [3]. Since the 2009 H1N1 influenza pandemic, associated academic societies have built ECMO centers and initiated ECMO education courses. As a result of these efforts, by 2016, the survival rate of H1N1 influenza respiratory patients on ECMO had increased, reaching the world standard of the ECMO center level [6].

Hence, based on the experience gained with H1N1 influenza ECMO, we are attempting to save the lives of as many COVID-19 severe respiratory failure patients as much as possible by spreading awareness about the appropriate use of ECMO with the help of the COVID-19 ECMOnet group.

## Data Availability

The datasets used and analyzed during the current study are available from the corresponding author on reasonable request.

## Abbreviations

ECMO: Extracorporeal membranous oxygenation
COVID-19: Coronavirus-induced disease 2019
